# Concentrations of the Stress Hormone Copeptin Increase upon Hypoglycaemia in Patients with Type 1 Diabetes Dependent of Hypoglycaemia Awareness

**DOI:** 10.1371/journal.pone.0072876

**Published:** 2013-08-30

**Authors:** Eleonora Seelig, Stefan Bilz, Ulrich Keller, Fabian Meienberg, Mirjam Christ-Crain

**Affiliations:** 1 Department of Diabetology, Endocrinology and Metabolism, University Hospital Basel, Basel, Switzerland; 2 Department of Endocrinology, Kantonsspital St. Gallen, St. Gallen, Switzerland; Pennington Biomedical Research Center, United States of America

## Abstract

**Objective:**

Copeptin, a marker for stress mirroring vasopressin concentrations, has been shown to increase upon insulin-induced hypoglycaemia in patients after transsphenoidal surgery of pituitary adenomas. Patients with type 1 diabetes mellitus are prone to hypoglycaemia, but no data about copeptin levels upon hypoglycaemia are available. Furthermore, the perception of hypoglycaemia can vary from total unawareness to disabling episodes. The aim of this study was to investigate whether copeptin increases upon hypoglycaemia in patients with type 1 diabetes mellitus and is associated with the degree of hypoglycaemia awareness.

**Materials and Methods:**

In this prospective observational study, 17 patients with type 1 diabetes underwent a standardized insulin infusion test. Blood sampling for glucose and copeptin was performed at baseline and after 60 minutes (min). To assess hypoglycaemia associated symptoms the Mood and Symptom Questionnaire (MSQ) was conducted at baseline and after 60 min.

**Results:**

During insulin infusion, blood glucose decreased from 5.1 (SD±0.2) to 3.0 (±0.5) mmol/L at 60 min (p<0.001). Copeptin concentrations increased from 3.2 (±1.7) to 3.8 (±1.9) pmol/L (p = 0.03). Mood and Symptoms Questionnaire scores increased from 14 (±3.0) to 18 (±5.8), (p = 0.006). Patients with good hypoglycaemia awareness had an increase in copeptin from 3.0 (±1.8) to 4.2 (±2.4) pmol/L (p = 0.03) in contrast to patients more unaware of hypoglycaemia who only showed an increase in copeptin from 3.3 (±1.6) to 3.6 (±1.4) pmol/L (p = 0.4). There was a trend to a larger copeptin increase in patients aware of hypoglycemia compared to patients unaware of hypoglycemia (p = 0.074).

**Conclusion:**

Copeptin increases in patients with type 1 diabetes upon insulin induced hypoglycaemia. Interestingly, the copeptin increase seems associated with the degree of hypoglycaemia awareness. This hypothesis warrants further verification.

**Trial Registration:**

ClinicalTrials.gov NCT00515801

## Introduction

Hypoglycaemia is an acute complication of insulin therapy. Patients with type 1 diabetes are particularly prone to develop hypoglycaemia. Physiologically, a strong stress reaction is activated during hypoglycaemia to counteract the hypoglycaemic state. In healthy subjects this stress reaction consists of a down-regulation of insulin secretion, followed by the secretion of hormones such as glucagon, epinephrine, growth hormone, adrenocorticotropin and vasopressin [1], [2].

In type 1 diabetes these neuroendocrine response mechanisms are often compromised and perception of hypoglycaemia is impaired. The secretion of insulin and glucagon deteriorates and the response of epinephrine is often delayed or blunted [3]. It seems that the pituitary hormone vasopressin also plays a role in the counter regulation probably by influencing the adrenocorticotropin secretion [4], [5]. Recently, copeptin, a glycopeptide representing the C-terminal part of the vasopressin prohormone, has emerged as a representative marker of stress. Several studies established the performance of this novel marker in situations such as sepsis, pneumonia, stroke and myocardial infarction. Its immediate up regulation in situations of stress even allowed diagnostic and prognostic conclusions [6]–[9]. In patients after transsphenoidal surgery, copeptin levels were shown to increase during insulin-induced hypoglycaemia [10]. However, the performance of copeptin during hypoglycaemia in patients with type 1 diabetes still needs to be elucidated.

The objective of this proof of concept study was to analyse the response of copeptin to hypoglycaemia in type 1 diabetes. Furthermore, we evaluated the association between copeptin response and hypoglycaemia awareness.

## Materials and Methods

The protocol for this trial and supporting CONSORT checklist are available as supporting information; see Checklist S1 and Protocol S1.

In this prospective observational study, a total of 17 patients with diabetes mellitus type 1 were included. Inclusion criteria were age 18 to 50 years and stable metabolic control (HbA1c levels <8.0%, and without history of severe hypoglycaemia in the past four weeks).

The study protocol was approved by the institutional review board (Human Ethics Committee of Basel). Written informed consent was obtained from all participating patients. The study took place at the University Hospital Basel.

All patients were admitted the night before beginning of the insulin infusion study. They were instructed to omit their basal insulin. Overnight blood glucose levels were adjusted between 5 and 8 mmol/l with a continuous intravenous insulin infusion with human insulin. Patients remained fasting until the end of the study. The next morning blood glucose levels were stabilized at 5 mmol/l one hour before the onset of the intravenous insulin infusion test. Thereafter the intravenous insulin infusion study was started. A standardized infusion with human insulin 30 mU/m^2^/min was administered for the duration of 60 min. Blood samples for glucose and copeptin measurements were obtained at baseline and after 60 min. Plasma glucose levels were assessed using an automated glucose analyser (2300 STAT Plus, YSI Bioanalytical Products). Copeptin, a secondary endpoint of the study, was measured using the CT-pro AVP LIA (Brahms AG, Hennigsdorf) as described elsewhere [11]. Hypoglycaemia was defined as plasma glucose ≤3.9 mmol/l, according to the guidelines of the American Diabetes Association. Hypoglycaemia associated symptoms were assessed at baseline and after 60 min using the Mood and Symptom Questionnaire (MSQ) [12]. The MSQ consists of 12 questions inquiring physical symptoms and mood, every question is rated on a 7-point scale (1 = no symptom, 7 = maximal symptoms). According to the MSQ score at 60 min and at baseline patients were stratified into patients with poor hypoglycemia awareness (MSQ at 60 min minus MSQ at baseline equal or below the median) and patients with good hypoglycemia awareness (MSQ at 60 min minus MSQ at baseline above the median).

Continuous variables are presented as mean (±standard deviation). Comparisons within subjects (at baseline and 60 minutes) were analysed using the paired t-test. Comparisons across subjects (unaware versus aware diabetics) were analysed using the unpaired t-test. The Kolmogorov-Smirnov test was applied to test normal distribution. Univariate and multivariate logistic regression models were used to analyse variables influencing copeptin increase. All tests were two tailed; p<0.05 was defined as significant. Data were analysed using statistical software (Statistical Package for Social Sciences, IBM SPSS Version 20, Chicago IL).

## Results

**Figure 1:**
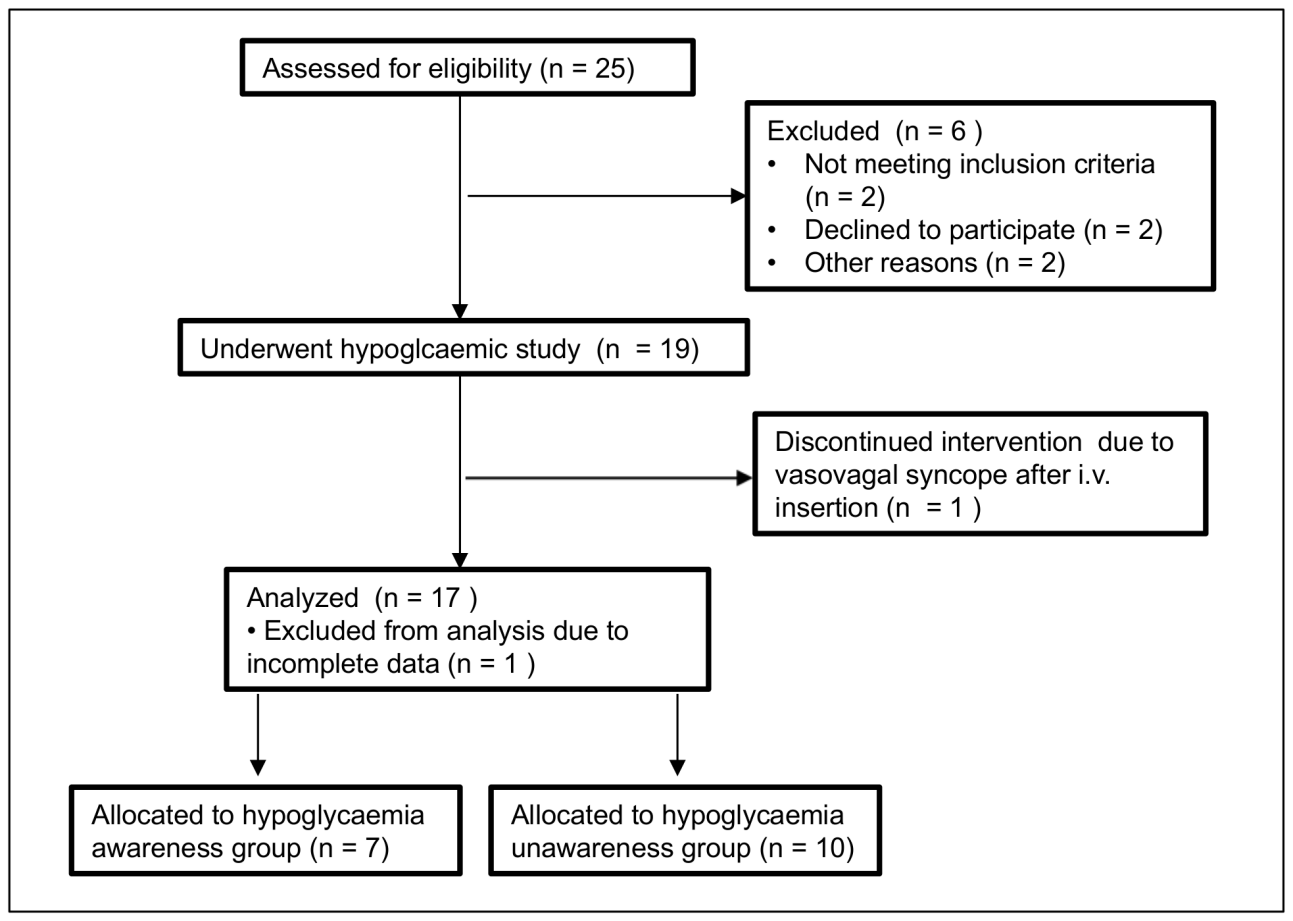
Recruitment of participants.

17 patients with diabetes mellitus type 1 (5 female, 12 male), mean age 39.6 years (standard deviation±10.1), mean diabetes duration 13.1 years (±12.5) and mean HbA1c level 7.3% (±1.1) were included in the study (Fig. 1).

**Figure 2:**
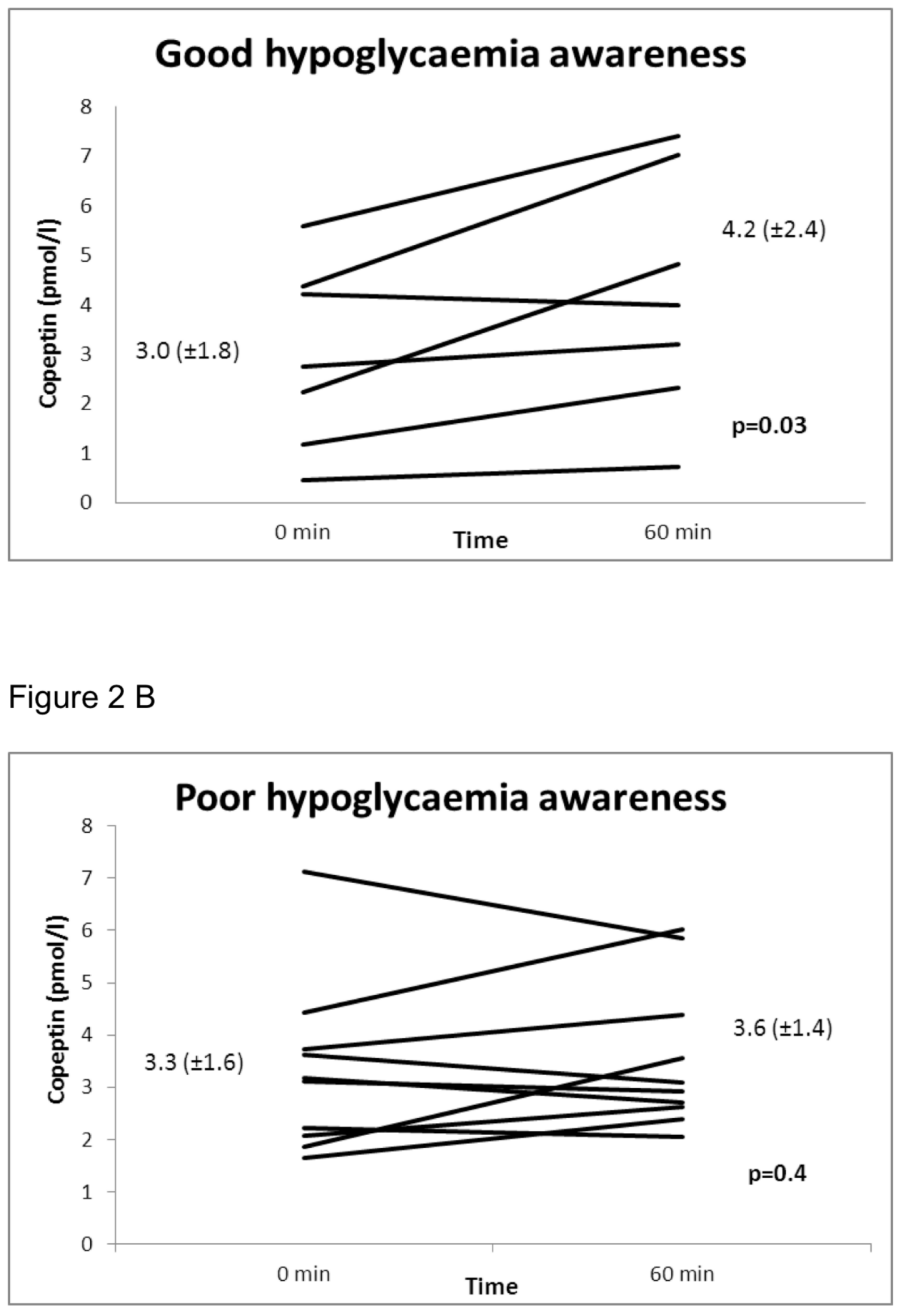
Copeptin and Hypoglycaemia Awareness. The increase of copeptin levels in patients with type 1 diabetes during hypoglycaemia depends on hypoglycaemia awareness. Patients more aware of hypoglycaemia (2a) had a significant increase in copeptin levels in contrast to patients with poor hypoglycaemia awareness (2b). Continuous variables are presented as mean (±standard deviation). Within group comparisons were analysed using paired t-test. Data were analysed using statistical software (Statistical Package for Social Sciences, IBM SPSS Version 20, Chicago IL).

During insulin infusion, blood glucose levels decreased from a mean of 5.1 (±0.2) at baseline to 3.0 (±0.5) mmol/L at 60 min (p<0.001). The mean copeptin levels increased from 3.2 (±1.7) to 3.8 (±1.9) pmol/L (p = 0.03), whereas mean MSQ scores increased from 14 (±3.0) to 18 (±5.8) points (p = 0.006). Patients with poor hypoglycemia awareness, had an increase in copeptin levels from 3.3 (±1.6) to 3.6 (±1.4) pmol/L (p = 0.4), whereas patients with good hypoglycemia awareness had an increase from 3.0 (±1.8) to 4.2 (±2.4) pmol/L (p = 0.03) (Figure 2a, 2b). Comparing both groups, revealed a trend for a copeptin increase in the latter group (p = 0.074). Similarly, in logistic regression there was a trend for hypoglycemia awareness (poor vs. good) in predicting copeptin increase (p = 0.074). Importantly, this trend was neither modified by gender nor HbA1c (copeptin p = 0.072 and 0.66, accordingly). There was no difference between patients aware and unaware of hypoglycemia concerning diabetes duration (8.6±11.5 vs. 16.3±12.8 years; p = 0.2) and HbA1c (7.6±0.9 vs. 7.1±1.3%; p = 0.4).

## Discussion

To our knowledge this is the first study investigating copeptin in patients with type 1 diabetes mellitus upon insulin induced hypoglycaemia. Herein, we demonstrate a copeptin increase during hypoglycemia in type 1 diabetics. Interestingly, this finding was restricted to patients with good hypoglycaemia awareness.

It is well known that hypoglycaemia activates the hypothalamic pituitary axis. Among many neuronal and humoral responses, neurons of the supraoptic and paraventricular nuclei are activated, subsequently releasing vasopressin [13]. The release of vasopressin in patients with and without diabetes during hypoglycaemia has been documented previously [2] [4], [14]. Noteworthy, the measurement of vasopressin is rather cumbersome since it is unstable in isolated plasma and has a tendency to aggregate [15], [16]. In contrast, copeptin, a cleavage product from the precursor peptide of vasopressin, has a long half-life ex vivo providing more reliable measurements. As copeptin is secreted in equimolar ratio to vasopressin, it offers an excellent alternative to the assessment of vasopressin [17].

In our study of patients with type 1 diabetes we were able to illustrate an increase of copeptin during hypoglycaemia. These findings agree with the results of previous studies [4], [14] where the neuroendocrine response of vasopressin to hypoglycaemia was enhanced in patients with type 1 diabetes compared to healthy subjects. Beside alterations of hypoglycaemia response mechanisms, patients with type 1 diabetes are likely to develop hypoglycaemia unawareness. It is assumed that recent preceding hypoglycaemic events are the origin of both, impaired counterregulation and hypoglycaemia unawareness [18]. However, exact mechanisms leading to hypoglycaemia unawareness still need to be explored. An impairment of the neuroendocrine response as a potential explanation is under discussion. To further elucidate the role of the neuroendocrine mechanisms we investigated the performance of copeptin in relation to hypoglycaemia unawareness. Interestingly, we could identify a copeptin increase only in patients with more hypoglycaemia symptoms, whereas in patients unaware of hypoglycaemia symptoms this increase was not evident. There was only a trend in a different copeptin increase when comparing patients with good and poor hypoglycaemia awareness. Potential confounders such as gender and HbA1c did not modify this trend. This novel, hypothesis-generating results indicate that vasopressin counter regulation might be associated with the development of hypoglycaemia unawareness in type 1 diabetes. Importantly, some limitations need to be considered. Due to the complex study procedures the sample size was small and powered for “within subject analyses” rather than “across subject analyses.” Consequently, comparisons between groups were underpowered. Induced hypoglycaemia in this study was only moderate. Since our study lacks a control group without diabetes results are restricted to patients with type 1 diabetes.

Summarized, copeptin increases in patients with type 1 diabetes upon insulin induced hypoglycaemia. Furthermore, vasopressin regulation may be associated with hypoglycaemia awareness in type 1 diabetics. Importantly, due to the small sample size this hypothesis requires further verification in a larger population.

## Supporting Information

Checklist S1 **CONSORT Checklist.**
(DOC)

Protocol S1 **Trial Protocol.**
(DOC)
