# Supplementary material for: Concentrations of the Stress Hormone Copeptin Increase upon Hypoglycaemia in Patients with Type 1 Diabetes Dependent of Hypoglycaemia Awareness
Source: PLoS One. 2013 Aug 30;8(8):e72876. doi: 10.1371/journal.pone.0072876 (PMC3758334; doi:10.1371/journal.pone.0072876)
Supplement: Protocol S1 — Trial Protocol. (DOC) [file pone.0072876.s002.doc]

Effect of a Sulfonylurea Compound on the Glucagon Response to Insulin-induced Hypoglycemia in Type 1 Diabetes Mellitus

*Eleonora Seelig, Karin Hegar, Fabian Meienberg, Lilly Linder, Ulrich Keller and Stefan Bilz*

*Division of Endocrinology, Diabetes and Clinical Nutrition, University Hospital Basel, 4031 Basel, Switzerland*

**Primary Investigator:**

Stefan Bilz, MD

Endocrinology, Diabetes and Clinical Nutrition

University Hospital Basel

Petersgraben 4,

4031 Basel, Switzerland

Phone: +41 61 265 5077

Fax: +41 61 265 5077

Email: [sbilz@uhbs.ch](mailto:sbilz@uhbs.ch)

Location, date: Basel, 12.02. 2007 Signature:
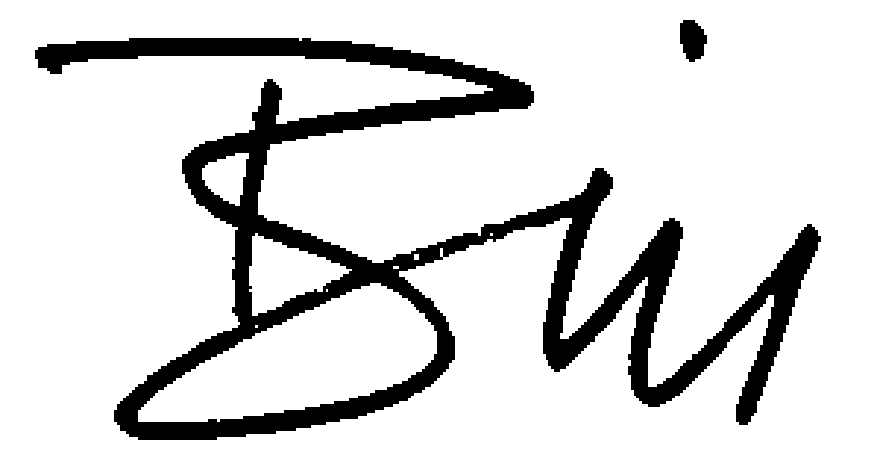


Table of Contents

[1. Introduction 2](#__RefHeading___Toc158014688)

[2. Study objectives 3](#__RefHeading___Toc158014689)

[3. Study design and methods 3](#__RefHeading___Toc158014690)

[3.1. General description of the study: 3](#__RefHeading___Toc158014691)

[3.2. Study population 3](#__RefHeading___Toc158014692)

[3.3. Study schedule 4](#__RefHeading___Toc158014693)

[3.4. Study medication 6](#__RefHeading___Toc158014694)

[3.5. Potential risks and adverse events 6](#__RefHeading___Toc158014695)

[3.6. Statistical Considerations 8](#__RefHeading___Toc158014696)

[4. Organisation 8](#__RefHeading___Toc158014697)

[4.1. Specific Location: 8](#__RefHeading___Toc158014698)

[4.2. Time schedule: 9](#__RefHeading___Toc158014699)

[5. Informed Consent, Ethical Review, Liability, Regulatory and Economic Considerations 9](#__RefHeading___Toc158014700)

[5.1. Informed consent 9](#__RefHeading___Toc158014701)

[5.2. Ethical review 9](#__RefHeading___Toc158014702)

[5.3. Liability 9](#__RefHeading___Toc158014703)

[5.4. Regulatory Considerations 9](#__RefHeading___Toc158014704)

[5.5. Economic Considerations 9](#__RefHeading___Toc158014705)

[6. Study investigators 10](#__RefHeading___Toc158014706)

[7. References 11](#__RefHeading___Toc158014707)

# Introduction:

Hypoglycemia is considered a limiting factor for insulin therapy in patients with diabetes mellitus type 1 and advanced diabetes type 2. Insulin-induced hypoglycemia not only causes symptomatic and sometimes temporarily disabling episodes but can be fatal. It also precludes the maintenance of euglycemia over a lifetime of diabetes and thus full realization of the benefits of glycemic control.

In healthy subjects the induction of hypoglycemia is followed by a coordinated hormonal response aimed at restoring normal blood glucose levels (1). Hypoglycemia results in suppression of endogenous insulin secretion from pancreatic β-cells and in increased secretion of counter regulatory hormones with glucagon and epinephrine being the first line response. All three of these physiologic defences may be defective in patients with type 1 diabetes rendering them at even increased risk for severe hypoglycemia (2). The lack of an adequate glucagon response to hypoglycemia occurs early in the course of type 1 DM and was first described by Gerich et al more than 30 years ago (3). In contrast to the defective epinephrine response, which is partially restored when hypoglycemic episodes are avoided, the impairment of the glucagon response is largely irreversible (4). The cessation of the adrenomedullary epinephrine response further promotes hypoglycemia-associated autonomic failure and hypoglycemia unawareness, thereby establishing a vicious cycle (2).

The most important stimulus for the pancreatic α-cell to secrete glucagon in response to decreasing glucose concentrations has been found to be the resulting decrement in β-cell insulin secretion. This has been concluded from studies demonstrating that both tonic stimulation of β-cell insulin secretion by sulfonylureas and tonic inhibition of β-cell insulin secretion by diazoxide impair the glucagon response to insulin induced hypoglycemia in healthy volunteers (5;6). According to this “insulin switch-off hypothesis” the impaired glucagon response during hypoglycemia in type 1 diabetes can be attributed to the intra-islet insulin deficiency and the resulting absence of a decrease in intra-islet insulin during hypoglycaemia (6).

Sulfonylureas, which are widely prescribed for the oral therapy of type 2 diabetes, act on ATP sensitive potassium channels (K_ATP_), also referred to as sulfonylurea receptor or SUR1/Kir6.2. Binding of sulfonylureas to pancreatic β-cell K_ATP_ channels results in closure of the channels, membrane depolarisation, calcium influx and exocytosis of insulin containing secretory granules.

The same K_ATP_ channels have recently been identified on glucagon secreting pancreatic α-cells (7). Inactivation of K_ATP_ channels in isolated α-cells by sulfonylureas resulted in enhanced glucagon secretion and results from islets isolated from SUR1 knockout mice suggest an important role for α-cell K_ATP_ channels in the regulation of glucagon secretion (8-10). These results appear contradictory to those previously obtained in normal subjects and in patients with type 2 DM in whom sulfonylureas have been reported to suppress glucagon concentrations during insulin-induced hypoglycemia (11;12). However, a recent study performed type 1 diabetics without residual β-cell function reported an increase of plasma glucagon concentrations following oral application of glibenclamide, a sulfonylurea drug (13).

These results suggest that α-cell K_ATP_ channels are important regulators of glucagon secretion in patients with type 1 diabetes without residual β-cell function. In this specific condition, the absence of intra-islet insulin may unmask direct stimulatory effects of sulfonylureas on α-cell glucagon secretion. Therefore, we hypothesize that inactivation of α-cell K_ATP_ channels by glibenclamide, a sulfonylurea compound, restores the defective glucagon response to insulin induced hypoglycemia in type 1 diabetics without residual β-cell function, as indicated by unmeasurable C-peptide concentrations. Consequently, sulfonylureas, which are widely used as hypoglycemic agents in type 2 diabetes, may turn out to be useful tools in the prevention of insulin-induced hypoglycemia in patients with type 1 diabetes.

# Study objectives:

To demonstrate that oral administration of glibenclamide stimulates pancreatic glucagon secretion during hypoglycemia in insulin-deficient (C-peptide negative) patients with type 1 diabetes when compared to type 1 diabetic patients with residual insulin secretion (C-peptide positive).

# Study design and methods:

## General description of the study:

Patients with type 1 diabetes participating in this single-blind, randomized cross-over study evaluating the effect of glibenclamide on glucagon secretion during insulin induced hypoglycemia. Glucose and glucagon metabolism will be studied using an intravenous insulin infusion test. We anticipate that patients diagnosed with C-peptide negative type 1 diabetes will increase glucagon secretion after oral administration of glibenclamide compared to patients with type 1 diabetes with maintained residual β-cell function.

## Study population:

Study subjects will be recruited according to the following criteria:

*Inclusion criteria for patients with type 1 diabetes*:

- Patients aged 18 to 50 years
- Patients diagnosed with C-peptide negative diabetes type 1 (C-peptide <200 pmol/L 6 min after 1 mg glucagon i.v. at plasma glucose concentrations between 5 and 11 mmol/l)
- Patients diagnosed with C-peptide positive diabetes type 1 (C-peptide > 500 pmol/l 6 min after 1 mg glucagon i.v. at plasma glucose concentrations between 5 and 11 mmol/l)
- Stable metabolic control; HbA1c levels <8.0 % and without episodes of antecedent severe hypoglycemias in the past four weeks

*Exclusion criteria*:

- Patients treated with medications potentially interfering with glucose metabolism, such as systemic steroids, immunosuppressive drugs (cyclosporine, tacrolimus, sirolimus), highly active antiretroviral therapy
- History coronary artery disease
- History of epilepsy or seizures
- Current smokers
- Any significant or unstable hepatic, cardiac, pulmonary, renal, neurological, musculoskeletal, hematological or endocrine disease.
- Pregnant or breast feeding women
- Woman of childbearing potential not using a reliable method of birth control such as oral contraceptives or IUD.
- Subjects refusing or unable to give written informed consent

## Study schedule:

### Patient recruitment:

Patients diagnosed with type 1 diabetes mellitus attending the outpatient clinic of the Division of Endocrinology, Diabetes and Clinical Nutrition at the University Hospital Basel will be asked to participate. Those deemed eligible will be given a detailed explanation of the study protocol. After written informed consent has been obtained, they will be invited to join a screening visit in our outpatient department. A total of 20 patients (10 patients per group) will be recruited.

### Screening visit

Patients eligible to participate will be asked to discontinue or taper long-acting insulin up to 24 h before the screening visit depending on the individual’s regimen. Short acting insulin will be used according to the patients’ individual regimen to keep plasma glucose concentrations within desired limits (5-11 mmol/l). All patients will be admitted to the outpatient clinic of the Division of Endocrinology of the University Hospital Basel (Petersgraben 4, Klinikum 2, ground floor) after fasting over night at 8 a.m. for approximately 90 min. A brief physical exam (including, blood pressure, heart rate, body weight and height, waist circumference) will be performed, and a baseline blood sample (including WBC, RBC, creatinine, electrolytes, C-peptide, HbA1c will be obtained). A pregnancy test will be obtained in women of childbearing potential.

Residual β-cell function will be assessed by measuring plasma C-peptide concentrations in the basal state and 6 min after the intravenous administration of 1 mg glucagon (GlucaGen Novo Nordisk® 1 mg ampules) as previously described (14;15). Subjects with a C-peptide response < 200 pmol/l to intravenous glucagon will be considered as “C-peptide negative”. The glucagon stimulation test will only be performed at fasting glucose values between 5 and 11 mmol/l in order to receive reproducible test results and will be rescheduled otherwise.

*Assessment of autonomic neuropathy and hypoglycemia awareness:*

Participants will be screened for the presence of autonomic neuropathy by the assessment of heart rate variability through ECG recording during deep inspiration and expiration, a Valsalva manoeuvre and orthostatic testing. A baseline 12-lead ECG will be obtained in addition.

Hypoglycemia awareness will be assessed by the hypoglycemia awareness questionnaire (HAQ) and the Mood and Symptom Questionnaire (MSQ)(16;17). Additionally, patients will be asked to keep a diary for 7 days after inclusion into the study protocol overlooking their blood glucose level monitored at least four times daily in correlation with possible hypoglycemia associated symptoms.

### Intravenous insulin infusion study 1:

Patients eligible to participate in the study protocol based on the results of the screening visit will be asked to discontinue or taper long-acting insulin at least 24 h before the intravenous insulin infusion study. Short acting insulin will be prescribed on an individual basis in order to keep capillary glucose concentrations between 5 and 11 mmol/l. All patients will be admitted to the Clinical Research Center, University Hospital Basel (Petersgraben 4, Klinikum 2, 6^th^ floor) between 8 and 9 p.m. for approximately **22 hours**, including an overnight stay. They will be asked to remain fasted after their regular dinner between 6 and 7 p.m. on the day of admission until the end of the insulin infusion study. **Patients will be under close supervision of a study physician throughout their inpatient stay.**

Two intravenous catheters will be inserted in antecubital veins and after a baseline blood sample has been obtained, an intravenous infusion of regular human insulin (10 Units Actrapid HM® in 50 ml normal saline) in will be started and continued overnight by means of a syringe pump (Braun perfusor® compact S). Adjustments based on blood glucose measurements every 15 to 60 min will be made to maintain blood glucose concentrations between 5 -8 mmol/l until the beginning of the experiment the next morning. The blood glucose will be stabilized at 5 mmol/l one hour before the onset of the intravenous insulin infusion study (see below). The intravenous line will be kept patent by infusion of a small amount of isotonic (0.9%) saline.

*Application of study drugs:*

At 7.30 a.m. all patients will receive 15 mg glibenclamide (3 tablets Daonil® 5 mg, Sanofi-Aventis Schweiz AG, 1217 Meyrin, Switzerland) or 3 placebo capsules (Doralgan Tabletten, Institute of Pharmacy, University Hospital Basel, 4031 Basel, Switzerland) with 200 ml of tap water in a randomized order. Glibenclamide plasma concentrations can be expected to peak ~ 2 hours after oral ingestion (18). Thereby, peak plasma concentrations will coincide with the glucose nadir during the insulin infusion study (see below).

*Randomisation procedure:*

A web-based random number generator ([www.random.org](http://www.random.org/)) will be used to assign a random number between 1 and 10 to each patient at the beginning of the first insulin infusion study. Those assigned an even number will receive glibenclamide during the first insulin infusion study and placebo pills during the second study. Patients assigned an odd number will receive the 2 therapies in reverse order.

*Insulin infusion and blood sampling:*

The intravenous insulin infusion test will be started at 8:30 a.m.. Regular human insulin will be infused at the rate of 30 mU/m²/min for 60 min and 15 mU/m²/min thereafter from 60 to 150 min in type 1 diabetics with a syringe pump (Braun perfusor® compact S) as previously described (19).. Venous blood samples will be taken at -30, -15, 0 min and at 5-10-min intervals over the next 150 min for the measurement of plasma glucose and at 15 min intervals for the measurement of plasma glucagon, epinephrine, metanephrine, growth hormone and cortisol concentrations. At the completion of the intravenous insulin study, the insulin infusion will be stopped, and 20% dextrose solution will be infused to re-establish normal glucose levels (4-8 mmol/l). The glucose infusion will be stopped when plasma glucose has stabilized >5 mmol/l for 30 min and a lunch will be served along with s.c. short acting insulin as needed. A total of 150 ml blood will be drawn during a metabolic stud

***Restoration of s.c. insulin therapy and glucose monitoring***

**After completion of the insulin infusion study the patient’s glucose levels will be monitored every 30-60 minutes until 10 hours after the ingestion of the study drug. They will be discharged from the Clinical Research Center at 5:30 p.m.. The usual insulin regime will be resumed at a reduced dose for 24 hours (70% of the usual dose, see also below) to account for the potential increase in endogenous insulin secretion elicited by glibenclamide. If blood glucose values increase above 10 mmol/l using this approach, small dosages of short-acting insulin will be applied to decrease blood glucose levels to the desired range.**

*Assessment of hypoglycemia associated symptoms:*

To assess hypoglycemia associated symptoms, the Mood and Symptom Questionnaire (MSQ), the Complex Reaction Time Task (CRTT) and the Paced Auditory Serial Addition Task (PASAT) will be performed at baseline and every 30 minutes until restoration of euglycemic glucose levels (17;20;21).

The CRTT has been developed to measure sustained visual attention and psychomotor speed. During a 5 minute period participants have to respond to the presentation of coloured lights (which appear in random sequence) by pressing corresponding buttons as accurately and fast as possible. Using a PC based control algorithm the inter-stimulus intervals is shortened or lengthened, thereby modifying task difficulty so that subjects’ false response rate within a continuously moving window approaches 50 percent (20).

A computerized version of the PASAT will be employed as a measure of sustained and divided attention and executive function of the working memory. Pacing will be at 2.5 s intervals, total task duration will be 180 s. Subjects will be instructed to sum up the last to digits presented as fast and accurate as possible. Accuracy and verbal response time will be assessed. Several forms of the test will be used as to prevent simple learning effects (21).

*Safety measures:*

The subjects’ consciousness during the insulin infusion study will be rated using the Glasgow coma scale (GCS) rating system every 5 min after plasma glucose has dropped below 3.5 mmol/l. If the grading decreases below 3 for best eye response (eye opening to speech), 3 for best verbal response (inappropriate words), 4 for best motor response (withdraws from pain) or plasma glucose concentrations decrease below 1.5 mmol/l, the insulin infusion will be stopped or tapered and small amounts of glucose (5-10g or 25-50 ml of a 20% dextrose infusion) will be given intravenously until the GCS rating and plasma glucose have increased above the previously mentioned limits.

Due to the prolonged duration of action of glibenclamide and its potential glucose lowering effects in type 1 diabetics with residual β-cell function, patients will be instructed to taper their usual insulin dose **by 30 %** and monitor their capillary blood glucose every two to four hours and any time after the onset of symptoms suggestive of hypoglycemia for 24 hours after oral application of glibenclamide. They will be asked to ingest 10 grams of oral dextrose if capillary blood glucose concentrations drop below 4.0 mmol/l. When ever correction of blood glucose levels < 4.0 mmol/l becomes necessary, a repeat glucose measurement will be performed after one hour. All participants will be provided with the phone number of a study investigator in case any problems should arise after discharge from the clinical research center. **All patients will be advised to refrain from vigorous physical activity and driving motor vehicles within 24 hours after the ingestion of the study drugs**. **Furthermore, patients will be advised to increase the dosage of short acting insulin if blood glucose readings exceed 10 mmol/l.**

### Intravenous insulin infusion study 2:

Patients will be asked to return for a second insulin infusion study within one month of the first study. All procedures will be identical as described above, except that subjects having received glibenclamide during the first insulin infusion study will receive placebo capsules and vice versa.

## Study medication:

Glibenclamide 5 mg tablets (Daonil® 5 mg, Swissmedic No. 35402, Lot-No. 40E108, expiration date 06.2008) has been purchased from Sanofi Aventis Schweiz AG, 1217 Meyrin, Switzerland.

Placebo tablets (Doralgan capsules, Lot-No. 060825N01, expiration date 08.2008) has been purchased from the Institute of Pharmacy at the University Hospital Basel, 4031 Basel, Switzerland.

Insulin Actrapid (Actrapid HM®, Swissmedic No. 44610, Lot-No. SW50921, expiration date 08.2008) has been purchased from Novo Nordisk Pharma AG, 8700 Küsnacht, Switzerland.

Glucagon 1 mg (GlucaGen Novo Nordisk® 1 mg ampules, Swissmedic No. 31489, Lot-No. SW60346, expiration date 01.2009) has been purchased from Novo Nordisk Pharma AG, 8700 Küsnacht, Switzerland.

## Potential risks and adverse events:

### Potential risks:

We consider the risks of this study to be mild. The specific risks are as follows:

Blood Loss: The total blood loss for this study will be approximately 300 ml.

Intravenous Catheters: Intravenous catheters used during the study are associated with a mild to moderate degree of pain upon insertion, and a small risk of localized infection.

Intravenous glucagon stimulation test: From our own experience the intravenous injection of glucagon is frequently associated with short-lasting (2-3 min) nausea and abdominal discomfort. Further side effects include vomiting, abdominal pain, paradoxic hypoglycemia, bradycardia (frequency 1:100 – 1:10’000) an rarely (frequency <1:10’000) with hypersensitivity, allergic reactions, hypoglycemic coma, tachycardia, hypotension, hypertension.

Intravenous insulin infusion: The intravenous insulin infusion is a safe technique which has been extensively used for the assessment of hypoglycemia counter regulation in type 1 diabetics (19). During the induction of hypoglycemia the subjects will be under close medical supervision. No long-term health effects can be anticipated from the induction of a short period of hypoglycemia. All infusions will be prepared under sterile conditions.

Insulin induced hypoglycemia following a bolus injection of regular human insulin (0.1-0.15 IU/kg body weight) is regularly used as a standard diagnostic procedure for the assessment of the hypothalamo-pituitary-adrenal axis and pituitary growth hormone secretory function at our institution. Venous plasma glucose concentrations between 1.5 and 2.0 mmol/l are regularly achieved during this procedure. Based on previous studies, the glucose nadir during our study can be anticipated to be around 2.0 mmol/l (40 mg/dl) (19). The insulin infusion will be discontinued or tapered if the plasma glucose concentration decreases below the threshold of 1.5 mmol/l (30 mg/dl) or significant impairment of consciousness occurs at any time point of the experiment (see section 3.3.3 – safety measures).

To minimize the potential risks associated with profound hypoglycemia, patients with a history of epilepsy, seizures and coronary disease are not eligible to participate. The upper age limit of 50 years and the exclusion of current smokers and patients with inadequate glycemic control (HbA1c > 8%) further minimizes the risk of silent coronary disease which may become clinically apparent during hypoglycemia.

Glibenclamide: Glibenclamide is a widely used drug for the treatment of type 2 diabetes. Possible adverse effects are headache, diarrhoea, constipation, nausea, vomiting or abdominal pain, allergic reactions such as skin rashes. Hypoglycemia due to the stimulation of β-cell insulin secretion is the most common side effect. Since β-cell function is severely impaired in type 1 diabetes, we do not expect significant insulin release following oral glibenclamide in the patients included in the study. Therefore, the risk of hypoglycemia will be minimal. Nevertheless, patients will be instructed to closely monitor their blood glucose levels and reduce their usual insulin dose by **30%** for up to 24 hours after glibenclamide treatment.

Placebo pills: No adverse effects can be anticipated from the intake of placebo pills.

### Adverse events:

The study investigators will oversee and monitor all aspects of the study including subject consent, study conduct, data handling as well as reporting any adverse events to the local ethical committee and Swissmedic

### Assessment of Risk:

The procedures included in this study are considered to present only mild risk to research participants. However, the study will be reviewed and reported as required by the “Verordnung über klinische Versuche mit Heilmitteln des Schweizerischen Bundesrates vom 17. Oktober 2001”.

### Attribution of Adverse Events:

Adverse events will be monitored for every subject participating in the study and attributed to the study procedures/design by the study investigators according to the following categories:

Possible: Adverse event(s) may be related to investigational procedure(s)/agent(s) or other intervention.

Unlikely: Adverse event(s) will doubtfully be related to investigational procedure(s)/agent(s) or other intervention.

### Plan for Grading Adverse Events:

The following grade levels will be used in defining the severity of adverse events:

Serious: Death, immediate risk of death, hospitalization or a prolongation of existing hospitalization or a persistent or significant disability/incapacity.

Non-serious: Mild or moderate adverse event or within normal limits.

### Plan for reporting serious adverse events:

While no serious adverse events are expected, the study investigators will report any serious adverse events to the local ethical committee and Swissmedic within 48 hours.

### Plan for reporting non-serious adverse events:

Non-serious unanticipated adverse events will be reported to the local ethical committee on an annual basis.

### Data and safety review and frequency:

The study investigators will review the data and study conduction on a weekly basis and determine whether the study should continue unchanged or whether modifications to the protocol and/or consent form are required.

## Statistical Considerations:

### Data analysis:

The primary endpoint for the comparison of patients with diabetes type 1 with and without treatment with glibenclamide will be the area under the curve of plasma glucagon concentrations from time 0 (start of the insulin infusion) to time 150 (150 min after the onset of the insulin infusion).

The secondary endpoint will be the rate of recovery of plasma glucose concentrations from 60 to 150 min, after tapering the insulin infusion to 15 mU/m^2^/min.

Further secondary endpoints will be the relation of symptoms and signs of hypoglycemia and hypoglycemia awareness to counter-regulatory hormone concentrations during insulin induced hypoglycemia.

The data analysis consists of the analysis of the primary and secondary endpoint. Data will be expressed as means ± SEM, Analysis of variance and appropriate post hoc analysis will be used to assess the effect of glibenclamide therapy over time. Unpaired t-tests and Mann-Withney U-tests will be used to compare baseline parameters between groups.

### Sample size calculation:

The sample size calculation for patients with C-peptide negative diabetes type 1 is based on ANCOVA comparing glucagon levels and an expected increase of 30% after glibenclamide treatment and α=0.05 using the following formula:

2[Zα+Zβ]^2^ [s] ^2^

= N

Δ^2^

where N is the required sample size, Zα and Zβ are the false positive and false negative error rates tolerates (Zα =1.96 (95%, 2 tailed); Zβ=0.84 (80% 1-tailed), s^2^ is the variance based on prior studies (square of the standard deviation (SD)) and Δ is the difference in the primary endpoint (change glucagon levels). The Glucagon level was found to be 22 ± 3 ng/l in patients with diabetes type 1 (13). The sample size will be 8. 10 patients per group will be recruited in order to account for potential drop-outs.

# Organisation:

## Specific Location:

Outpatient Clinic, Division of Endocrinology, Diabetes and Clinical Nutrition, University Hospital Basel, Klinikum 2, Petersgraben 4, Basel, Switzerland

Clinical Research Center, University Hospital Basel, Klinikum 2, Petersgraben 4, Basel, Switzerland.

## Time schedule:

Start of the study: March 2007

Duration: 9 months including recruitment period.

# Informed Consent, Ethical Review, Liability, Regulatory and Economic Considerations:

## Informed consent:

Subjects are required to provide written informed consent before enrollment into the study protocol. The consent includes a statement that enrolled subjects agree on the use of their data for scientific purposes. Patients who are not able to give written informed consent are not included in the study. The informed consent document will be used to explain in simple terms the aim of the study, required visits and procedures. The informed consent document contains a statement that the consent is freely given, and that the patient is free to withdraw from the study at any time.

## Ethical review:

This protocol will be submitted for approval to the conjoined local ethic committee of Basel and Baselland and to Swissmedic for approval of the use of glibenclamide (Daonil®) and placebo pills.

## Liability:

The study procedures will be covered by the general liability insurance RIMAS* of the University Hospital Basel, as will be certified by Jürg Müller (Rechtsdienst, University Hospital Basel).

(* RIMAS, Insurance-Broker AG, Hanspeter Kolmos, Fischmarkt 10, Postfach 1919, CH-4001 Basel, Tel.: 061 269 81 13, Fax: 061 269 81 10).

## Regulatory Considerations:

This study will be conducted in accordance with the ethical principles stated in the most recent version of the Declaration of Helsinki or the applicable International Conference on Harmonization (ICH) guidelines on good clinical practice, whichever represents the greater protection of the individual.

The study will not be started until approval of the study protocol by the local ethical committee and Swissmedic.

Data collected will be kept confidential and accessible only to researchers involved, the ethical review committee and Swissmedic. The data records will be stored in a locked room and the electronic data will be kept on a computer with password protection. All study records will be kept for at least 15 years.

## Economic Considerations:

No additional costs will arise through participation in the study, i.e. there are no cost to the patients or their health insurance. There will be no charges for any of the testing procedures during the study. Patients will be paid a total of CHF 250 .- for each completed inpatient study, i.e. a total of CHF 500.- for patients completing the study per protocol. Patients not eligible to participate based on the results of the screening visit will receive no imbursement.

The salary of E. Seelig is provided by the Lichtenstein-Stiftung Basel, Switzerland. Further financial support will be requested from the “Diabetesstiftung der Region Basel” and the “Wissenschaftlicher Kredit des Universitätsspitals Basel” and the Novartis-Fonds at the University Hospital Basel.

# Study investigators:

| **Name** | **Institution** | **Phone** | **Email** |
| --- | --- | --- | --- |
| **Stefan Bilz, MD** | Endocrinology, Diabetology and Clinical Nutrition, University Hospital Basel, Switzerland | 061 328 6077 | sbilz@uhbs.ch |
| **Eleonora Seelig, MD** | Endocrinology, Diabetology and Clinical Nutrition University Hospital Basel, Switzerland | 061 328 6814 | eseelig@uhbs.ch |
| **Prof. Ulrich Keller, MD** | Endocrinology, Diabetology and Clinical Nutrition, University Hospital Basel, Switzerland | 061 328 6073 | ukeller@uhbs.ch |
| **Lilly Linder, MD** | Endocrinology, Diabetology and Clinical Nutrition, University Hospital Basel, Switzerland | 061 328 6217 | Lilly.Linder@unibas.ch |
| **Karin Hegar, PhD** | Endocrinology, Diabetology and Clinical Nutrition, University Hospital Basel, Switzerland | 061 328 6802 | HegarK@uhbs.ch |
| **Fabian Meienberg, MD** | Endocrinology, Diabetology and Clinical Nutrition, University Hospital Basel, Switzerland | 061 265 2525 | fmeienberg@hotmail.com |

# References:

(1) Rizza RA, Cryer PE, Gerich JE. Role of glucagon, catecholamines, and growth hormone in human glucose counterregulation. Effects of somatostatin and combined alpha- and beta-adrenergic blockade on plasma glucose recovery and glucose flux rates after insulin-induced hypoglycemia. J Clin Invest. 1979;64:62-71.

(2) Cryer PE. Diverse Causes of Hypoglycemia-Associated Autonomic Failure in Diabetes. N Engl J Med. 2004;350:2272-79.

(3) Gerich JE, Langlois M, Noacco C, Karam JH, Forsham PH. Lack of Glucagon Response to Hypoglycemia in Diabetes: Evidence for an Intrinsic Pancreatic Alpha Cell Defect. Science. 1973;182:171-73.

(4) Dagogo-Jack S, Rattarasarn C, Cryer PE. Reversal of hypoglycemia unawareness, but not defective glucose counterregulation, in IDDM. Diabetes. 1994;43:1426-34.

(5) Banarer S, McGregor VP, Cryer PE. Intraislet hyperinsulinemia prevents the glucagon response to hypoglycemia despite an intact autonomic response. Diabetes. 2002;51:958-65.

(6) Raju B, Cryer PE. Loss of the Decrement in Intraislet Insulin Plausibly Explains Loss of the Glucagon Response to Hypoglycemia in Insulin-Deficient Diabetes: Documentation of the Intraislet Insulin Hypothesis in Humans. Diabetes. 2005;54:757-64.

(7) Rajan AS, Aguilar-Bryan L, Nelson DA, Nichols CG, Wechsler SW, Lechago J et al. Sulfonylurea receptors and ATP-sensitive K+ channels in clonal pancreatic alpha cells. Evidence for two high affinity sulfonylurea receptors. Journal of Biological Chemistry. 1993;268:15221-28.

(8) Franklin I, Gromada J, Gjinovci A, Theander S, Wollheim CB. {beta}-Cell Secretory Products Activate {alpha}-Cell ATP-Dependent Potassium Channels to Inhibit Glucagon Release. Diabetes. 2005;54:1808-15.

(9) Munoz A, Hu M, Hussain K, Bryan J, Aguilar-Bryan L, Rajan AS. Regulation of glucagon secretion at low glucose concentrations: evidence for adenosine triphosphate-sensitive potassium channel involvement. Endocrinology. 2005;146:5514-21.

(10) Hoy M, Olsen HL, Bokvist K, Buschard K, Barg S, Rorsman P et al. Tolbutamide stimulates exocytosis of glucagon by inhibition of a mitochondrial-like ATP-sensitive K+ (KATP) conductance in rat pancreatic A-cells. J Physiol (Lond). 2000;527:109-20.

(11) Landstedt-Hallin L, Adamson U, Lins PE. Oral Glibenclamide Suppresses Glucagon Secretion during Insulin-Induced Hypoglycemia in Patients with Type 2 Diabetes. Journal of Clinical Endocrinology Metabolism. 1999;84:3140-3145.

(12) ter Braak EWMT, Appelman AMMF, van der Tweel I, Erkelens DW, van Haeften TW. The Sulfonylurea Glyburide Induces Impairment of Glucagon and Growth Hormone Responses During Mild Insulin-Induced Hypoglycemia. Diabetes Care. 2002;25:107-12.

(13) Ostergard T, Degn KB, Gall MA, Carr RD, Veldhuis JD, Thomsen MK et al. The Insulin Secretagogues Glibenclamide and Repaglinide Do Not Influence Growth Hormone Secretion in Humans but Stimulate Glucagon Secretion during Profound Insulin Deficiency. Journal of Clinical Endocrinology Metabolism. 2004;89:297-302.

(14) Faber OK, Binder C. C-peptide response to glucagon. A test for the residual beta-cell function in diabetes mellitus. Diabetes. 1977;26:605-10.

(15) Palmer JP, Fleming GA, Greenbaum CJ, Herold KC, Jansa LD, Kolb H et al. C-peptide is the appropriate outcome measure for type 1 diabetes clinical trials to preserve beta-cell function: report of an ADA workshop, 21-22 October 2001. Diabetes. 2004;53:250-264.

(16) Clarke WL, Cox DJ, Gonder-Frederick LA, Julian D, Schlundt D, Polonsky W. Reduced awareness of hypoglycemia in adults with IDDM. A prospective study of hypoglycemic frequency and associated symptoms. Diabetes Care. 1995;18:517-22.

(17) Weinger K, Jacobson AM, Draelos MT, Finkelstein DM, Simonson DC. Blood glucose estimation and symptoms during hyperglycemia and hypoglycemia in patients with insulin-dependent diabetes mellitus. The American Journal of Medicine. 1995;98:22-31.

(18) Jonsson A, Chan JC, Rydberg T, Vaaler S, Hallengren B, Cockram CS et al. Effects and pharmacokinetics of oral glibenclamide and glipizide in Caucasian and Chinese patients with type-2 diabetes. Eur J Clin Pharmacol. 2000;56:711-14.

(19) Bolli GB, De Feo P, De Cosmo S, Perriello G, Ventura MM, Benedetti MM et al. A reliable and reproducible test for adequate glucose counterregulation in type I diabetes mellitus. Diabetes. 1984;33:732-37.

(20) Langewitz, W, Bieling, H, Stephan JA, and Otten, H. A new self adjusting reaction time device (BonnDet) with high test-retest reliability. J Psychophysiology 1, 67-77. 1987.

(21) Gronwall DM. Paced auditory serial-addition task: a measure of recovery from concussion. Percept Mot Skills. 1977;44:367-73.
